# Mental health and smoking cessation—a population survey in England

**DOI:** 10.1186/s12916-020-01617-7

**Published:** 2020-06-25

**Authors:** Leonie S. Brose, Jamie Brown, Ann McNeill

**Affiliations:** 1 Addictions, Institute of Psychiatry, Psychology and Neuroscience, King’s College London, 4 Windsor Walk, London, SE5 8BB UK; 2 http://spectrum.ac.uk/; 3 Research Department of Clinical, Educational and Health Psychology, University College London, 1-19 Torrington Place, London, WC1E 7HB UK; 4 Department of Epidemiology and Public Health, University College London, 1-19 Torrington Place, London, WC1E 7HB UK

**Keywords:** Tobacco smoking, Smoking cessation, Mental health, Electronic cigarettes

## Abstract

**Background:**

To reduce inequalities between individuals with and without mental health problems, a better understanding is required of triggers and success of quit attempts among the third of smokers with mental health problems. The aim was to assess whether there are differences by mental health status in (i) triggers for quit attempts, (ii) use of evidence-based support (iii) and quit success.

**Methods:**

Monthly cross-sectional household surveys of representative samples of the adult population in England. In 2016/2017, 40,831 adults were surveyed; 1956 who had attempted to stop smoking cigarettes in the past year were included. Logistic regressions assessed associations between mental health (ever diagnosis, past-year treatment, past-month distress), triggers, support used and quit success, adjusting for sociodemographic and smoking characteristics.

**Results:**

Concern about future health, current health problems and expense of smoking were the most common triggers overall. For respondents with an ever diagnosis, past-year treatment or serious past-month distress, quit attempts were more frequently triggered by current health problems. Non-evidence-based support and e-cigarettes were used most often, and this did not differ by mental health status. Respondents with an ever diagnosis and moderate or serious distress were less likely to have used non-prescription nicotine replacement therapy (NRT). Respondents with past-year treatment or serious distress were more likely to have used prescription medication/behavioural support. Quit success did not differ by mental health status. Compared with non-evidence-based support, non-prescription NRT conferred no benefit. There was some evidence that prescription medication/behavioural support was beneficial (depending on outcome and adjustment, ORs ranged from 1.46, 95% CI 0.92–2.31, to 1.69, 1.01–2.86). E-cigarettes were associated with higher success rates after adjustment for different indicators of mental health (ORs ranged from 2.21, 1.64–2.98, to 2.25, 1.59–3.18).

**Conclusions:**

Smokers with mental health problems were more likely to have attempted to quit because of health problems and were more likely to have used gold standard support (medication and behavioural support) than other smokers. E-cigarettes were strongly associated with increased success and were used similarly by those with and without mental health problems, indicating that improved uptake of e-cigarettes for smoking cessation among smokers with mental health problems could help address inequalities.

## Background

There are substantial inequalities in morbidity and premature mortality between individuals with mental health problems and those without. Much of this inequality is due to preventable risks such as smoking, excessive alcohol consumption, sleep disturbance, physical inactivity and dietary risks [1–4]. Tobacco control strategies have led to an overall decline in smoking prevalence in countries such as the UK and the United States of America (US); however, there remains a large gap in smoking prevalence between people with and without mental health problems. Among those with a common mental health disorder in England, smoking prevalence remains around 50% higher [5], and this increases further for more severe mental disorders [6]. Figures for the US also show an increased burden of smoking among those with mental health problems [7–9]. About one third of smokers have a mental health problem [6, 10], and the relationship between mental health and smoking is complex and appears to be bidirectional [4, 11, 12]. Previous research has consistently shown that smokers with mental health problems on average smoke more heavily [5–7, 10] and extract more nicotine from each cigarette than those without mental health problems [6].

Successful smoking cessation is associated with subsequently improved physical and mental health [4, 13], reduced financial stress and, for people on specific psychotropic medication, a reduction in dose and side effects [6, 11]. Surveys in the UK and the US have consistently found negative associations between having mental health problems and having quit smoking [5, 14, 15], although this association is attenuated when taking heaviness of smoking into account [5]. Increased smoking cessation among smokers with mental health problems would reduce significant avoidable costs to the economy, consisting of direct medical costs, lost productivity and premature mortality [16]. Thus, there is an urgent need to improve cessation in this group as emphasised by a cross-government outcomes strategy [17], the National Institute for Health and Care Excellence (NICE, [18]) and a Lancet Psychiatry Commission [4].

In order to reduce the personal and societal inequalities caused by smoking, each step of the quitting process needs to be addressed [19] to help increase the proportion of smokers with mental health problems who quit successfully. An increase in quitting can be achieved by increasing the frequency of quit attempts, e.g. by effectively triggering attempts, and by increasing the likelihood of success for each attempt by more frequent use of evidence-based support. We have recently shown that smokers with mental health problems in England were more motivated to stop smoking and more likely to attempt to stop than smokers without mental health problems [10]. However, less than one third had attempted to stop smoking in the past year, so further evidence is needed on approaches which may help increase attempts to stop smoking. Evidence for the effectiveness of support indicates that varenicline is more efficacious than bupropion or nicotine replacement therapy (NRT) which in turn are more effective than placebo [20–22] and that behavioural support in addition to pharmacological support generally improves outcomes [23], for those with and without mental health problems.

There is little evidence to date on the extent to which smokers with mental health problems use the most effective support [21] and how different types of support are associated with the success of attempts to stop smoking in the real world.

## Methods

### Aims

This study aimed to address three research questions:

1. What are the most frequent triggers for quit attempts in smokers who (a) have had a diagnosis of a mental health problem, (b) have had treatment for this mental health problem in the past year or (c) have experienced moderate or serious psychological distress in the past month? Does this differ from smokers without mental health problems?
2. Does the use of evidence-based smoking cessation support differ in smokers with and without (a) a diagnosis of a mental health problem, (b) past-year treatment or (c) past-month distress?
3. Does the success of quit attempts with different aids vary in smokers with and without (a) a diagnosis of a mental health problem, (b) past-year treatment or (c) past-month distress?

### Design and setting

The present study used self-reported data from monthly cross-sectional household surveys of representative samples of the population of adults in England collected as part of the ongoing Smoking and Alcohol Toolkit Study. The methodology for the Toolkit Study has been described in detail by Fidler et al. [24], and to date, it has provided data for approximately 85 peer-reviewed publications. Briefly, each month (wave), a new sample of approximately 1700 adults aged 16 years or older in England completes a face-to-face computer-assisted survey. After stratification by a geo-demographic classification of the population, small geographical areas containing approximately 300 households are allocated randomly. Interviewers visit households within the random locality and conduct computer-assisted face-to-face interviews with one member of a household in those areas until a pre-specified quota tailored to the area is fulfilled. This form of sampling has benefits over conventional quota sampling, because the allocation of small areas to interviewers reduces the impact of selection bias resulting from the selection of properties [25]. Response rates cannot be calculated because of the lack of a definitive gross sample: all units fulfilling the criteria of a given quota are interchangeable within the areas.

### Measures

For the present study, measures of mental health were added to the survey for 24 monthly waves from January 2016 to December 2017. Mental health information was self-completed by the interviewee on a laptop following familiarisation using similar example questions. All other questions were completed by the interviewer who prompted the interviewee for responses and recorded do not know/prefer not to say where needed.

*Mental health* was measured using three indicators: ever diagnosis, past-year treatment and past-month distress (moderate or serious, measured using the Kessler 6 (K6) scale [26–28]); full details are given in Table 1.

**Table 1:** Measures assessing mental health, triggers for cessation attempt, support used during and outcome of quit attempt

**Mental health measures**	
**Ever diagnosis**	
Since the age of 16, which of the following, if any, has a doctor or health professional ever told you that you had? [all that apply, presented in randomised order, excluding the final three options]	
a) Depression	
b) Anxiety	
c) Obsessive-compulsive disorder	
d) Panic disorder or a phobia	
e) Post-traumatic stress disorder	
f) Psychosis	
g) Personality disorder	
h) Attention deficit hyperactivity disorder	
i) An eating disorder	
j) Alcohol misuse or dependence	
k) Drug use or dependence	
l) Problem gambling	
m) None of these	
n) Do not know	
o) Prefer not to say	
Categorised as ever having had a diagnosis if yes to any of a) to l).	
**Past-year treatment**	
In the last 12 months, which of the following conditions, if any, have you had any treatment or taken any prescribed medication for? Response options were any conditions that had been selected in the previous question as well as none of these, do not know and prefer not to say.	
**Past-month distress**	
During the past 30 days, about how often, if at all, did you feel…	
a) Nervous	
b) Hopeless	
c) Restless or fidgety	
d) So depressed that nothing could cheer you up	
e) That everything was an effort	
f) Worthless	
For each, the respondent indicated one of the following: all of the time (scored 4), most of the time (3), some of the time (2), a little of the time (1) and none of the time (0).	
A sum score with a possible range from 0 to 24 was calculated; scores between 5 and 12 were categorised as moderate distress [28] and scores of 13 and higher as serious distress [26, 27].	
**Outcome measures**	
**Quit attempt triggers**	
Q632c3a. Which of the following do you think contributed to you making the most recent quit attempt? [all that apply, presented in randomised order, excluding the final option]	
a) Advice from a GP/health professional	
a) TV advert for a nicotine replacement product	
b) Government TV/radio/press advert	
c) Hearing about a new stop smoking treatment	
d) A decision that smoking was too expensive	
e) Being faced with smoking restrictions	
f) I knew someone else who was stopping	
g) Seeing a health warning on a cigarette packet	
h) Being contacted by my local NHS Stop Smoking Services	
i) Health problems I had at the time	
j) A concern about future health problems	
k) Attending a local stop smoking activity or event	
l) Something said by family/friends/children	
m) A significant birthday	
n) Others (please specify)	
**Support used**	
Q632e40. Which, if any, of the following did you try to help you stop smoking during the most recent serious quit attempt? [all that apply, presented in randomised order excluding the final option]	
a) Nicotine replacement product (e.g. patches/gum/inhaler) without a prescription	
b) Nicotine replacement product on prescription or given to you by a health professional	
c) Zyban (bupropion)	
d) Champix (varenicline)	
e) Attended a Stop Smoking group	
f) Attended one or more Stop Smoking one-to-one counselling/advice/support session/s	
g) Phoned a Smoking Helpline	
h) A book or booklet	
i) Visited www.nhs.uk/Smokefree website	
j) Visited a website other than Smokefree	
k) Used an application (‘app’) on a handheld computer (smartphone, tablet, PDA)	
l) Hypnotherapy	
m) Acupuncture	
n) Electronic cigarette	
o) Heat-not-burn cigarette (e.g. iQOS, heatsticks)	
p) Allen Carr Easyway session	
q) Allen Carr Easyway book	
r) The SmokeFree Formula book	
s) Other book or booklet	
t) Others (please specify)	
As outcome variable dummy-coded as (1) ‘non-evidence-based support’ (h, i, j, k, l, m, o, p, q, r, s, t), (2) ‘NRT over the counter’ (a), (3) ‘e-cigarettes’ (n) and (4) ‘prescription medication and/or behavioural support’ (b, c, d, e, f, g). Categorised hierarchically, so where respondents used more than one type of support, the type with evidence for higher effectiveness was recorded [29–31]. NRT over the counter was separated from prescription medication as NRT without support appears to be a weaker option [30–32]. Numbers were too small to separate group 4 further as initially planned.	
**Quit success**	
How long did your most recent serious quit attempt last before you went back to smoking?	
a) Still not smoking	
b) Less than a day	
c) Less than a week	
d) More than 1 week and up to a month	
e) More than 1 month and up to 2 months	
f) More than 2 months and up to 3 months	
g) More than 3 months and up to 6 months	
h) More than 6 months and up to a year	
If respondents were not smoking at the time of the survey (a), they were categorised as having made a successful attempt. This was restricted to those whose attempt started at least 1 month ago for some analysis.	

The *outcome measures* used were (1) triggers that prompted the most recent quit attempt, (2) the type of support used during the attempt and (3) the outcome of the quit attempt by the time of the survey (Table 1).

*Sociodemographic measures* included age (16–24, 25–34, 35–44, 45–54, 55–64, 65 and over), gender and occupational grade (AB: higher or intermediate managerial, administrative or professional occupation; C1: supervisory or clerical and junior managerial, administrative or professional; C2: skilled manual workers; D: semi-skilled and unskilled manual workers; E: state pensioners, casual and lowest grade workers, unemployed with state benefits only [33]).

*Other measures related to smoking and smoking cessation* asked of all respondents included smoking status (currently smoking daily, currently smoking non-daily, stopped smoking in the last year), type of cigarette (hand-rolled, manufactured, mix of both, unknown), strengths of urges to smoke (from 0 for no urges to 5 for extremely strong urges [29, 34]) and number of quit attempts in the past year. Those who had made at least one quit attempt were asked whether the most recent quit attempt was planned or not, whether they had attempted to stop abruptly or cut down to quit and how much time had passed since the quit attempt started (up to 1 month, 1 to 6 months and more than 6 months). The type of support used was also included as an independent variable in some analyses where it was coded hierarchically as a single variable (non-evidence-based support, NRT over the counter, e-cigarettes, prescription medication and/or behavioural support).

### Participants

In 2016 and 2017, 40,831 adults were surveyed of whom 7465 were past-year (current and recent-ex) cigarette smokers. Of those, 6280 provided answers to all mental health questions of whom 1993 had attempted to stop smoking or stopped smoking in the past year. Respondents who responded ‘do not know’ or ‘prefer not to say’ to any of the relevant measures were excluded, leaving *n* = 1956 for analyses addressing research questions 1 and 2. To address research question 3, the main analysis excluded those whose quit attempt had started less than 1 month ago, leaving *n* = 1639 respondents. Sensitivity analysis for research question 3 included the full sample used for research questions 1 and 2, with no restriction on time since the quit attempt began.

### Statistical analysis

The analyses plan for this and a related paper [10] were pre-registered on the Open Science Framework (https://osf.io/9pbv8/). Data were weighted to match the English population profile on age, social grade, region, tenure, ethnicity and working status within gender. The dimensions are derived monthly from a combination of the English 2011 census, Office for National Statistics mid-year estimates and an annual random probability survey conducted for the National Readership Survey. We calculated weighted descriptives for the sample and then ran analyses to address each of the research questions without the weights applied.

1. *Quit attempt triggers*: The percentage selecting each option as prompting the most recent quit attempt was calculated. For prompts reported by at least 5% overall, logistic regressions were conducted with each prompt as outcome regressed onto mental health status (separately for ever diagnosis, past-year treatment and past-month distress), first unadjusted, then adjusted for age, gender, occupational grade, urges to smoke and time since most recent quit attempt (collapsed into up to 6 months or more than 6 months ago), thus replicating and extending an earlier analysis [35]. Additional unplanned analyses also included the type of cigarette with results reported if associations differ from the main analyses.
2. *Use of support*: The percentage using each type of support during the most recent quit attempt was calculated, followed by logistic regression with each type of support (dummy-coded) regressed onto mental health status (separately for ever diagnosis, past-year treatment and past-month distress), first unadjusted, then adjusted for age, gender, occupational grade, urges to smoke, whether the quit attempt was abrupt or included cutting down first and whether it was a planned or unplanned quit. An additional unplanned analysis included the type of cigarette. Chi-square comparisons described in the analysis plan were omitted in favour of unadjusted logistic regressions.
3. *Quit success*: Successful quit—defined as currently not smoking following a quit attempt starting at least 1 month earlier—was regressed onto support used (coded as hierarchical single variable), mental health status (separately for ever diagnosis, past-year treatment and past-month distress), age, gender, occupational grade, urges to smoke, mean number of past-year quit attempts, type of cigarette (manufactured only versus roll-your-own, mix, unknown) and time since quit attempt started, using unadjusted and adjusted logistic regressions again replicating and extending an earlier analysis [29]. A final model consisted of the adjusted logistic regression with additional interaction terms for mental health status and support used. Sensitivity analyses in addition to the pre-specified analysis repeated the main analyses using abstinence from smoking after a quit attempt without restriction on the time since the quit attempt started to increase comparability with research questions 1 and 2 and other papers using the Smoking Toolkit Study data [29, 30, 36].

Where associations were not statistically significant, Bayes factors (BFs) were calculated to determine whether results were supportive of the null hypothesis, the alternative hypothesis, or insensitive to detect a difference. We used a conservative approach with the alternative hypothesis represented by a half-normal distribution. Expected effect size for mental health conditions was set to odds ratio (OR) = 0.64 based on previous studies that have suggested a negative association between mental health conditions and smoking cessation [5]. Expected effect sizes were set to 0.96 for NRT over the counter [29] and to 1.95 for e-cigarettes [30]. For prescription medication and/or behavioural support, in two separate calculations, smaller (1.61 [29]) and larger effect sizes (3.25 [29]) were used to reflect varied estimates in the literature. BFs > 3 can be interpreted as evidence for the alternative hypothesis (and against the null), BFs < 1/3 as evidence for the null hypothesis and BFs between 1/3 and 3 as evidence that the data are insensitive to distinguish the alternative hypothesis from the null.

## Results

Four in ten respondents (40.2%) reported ever having had a mental health diagnosis since the age of 16, 25.9% reported treatment for a mental health problem in the past year and 40% reported moderate or serious past-month distress (Table 2). For the prevalence of individual diagnoses, see Additional File 1, Table S1.

**Table 2:** Sample description overall and by ever diagnosis, past-year treatment and past-month distress, weighted

Characteristic		Total	Ever diagnosis		Past-year treatment		Past-month distress		
			No	Yes	No	Yes	None	Moderate	Serious
**Ever diagnosis, %**		40.2	–	–	0	100	21.4	59.2	90.6
**Past-year treatment, %**		25.9	–	64.5	–	–	9.9	39.6	75.3
**Past-month distress, %**	Moderate	28.3	19.3	41.6	23.1	43.3	–	–	–
	Serious	11.7	1.8	26.4	3.9	33.8	–	–	–
**Age, %**	16–24	19.0	18.2	20.2	18.6	20.1	14.4	25.3	27.5
	25–34	26.8	25.3	29.0	26.0	29.0	25.5	29.0	27.9
	35–44	20.2	19.9	20.7	20.1	20.7	19.9	21.2	19.7
	45–54	17.1	17.4	16.5	17.3	16.5	19.6	12.7	14.3
	55–64	10.2	10.7	9.4	10.2	10.0	11.6	8.0	8.6
	≥ 65	6.7	8.5	4.0	7.8	3.7	8.9	3.9	2.0
**Gender, %**^**1**^	Men	51.8	58.4	42.0	56.8	37.5	55.3	50.8	36.5
	Women	48.2	41.6	58.0	43.2	62.5	44.7	49.2	63.5
**Occupational grade, %**^**2**^	AB	17.9	20.4	14.2	19.8	12.4	20.1	16.4	10.3
	C1	25.1	26.0	23.7	26.6	21.1	26.7	23.7	20.6
	C2	25.4	28.0	21.7	27.0	20.9	25.8	26.3	21.8
	D	18.2	17.5	19.2	17.8	19.4	18.0	17.3	21.4
	E	13.3	8.0	21.1	8.8	26.1	9.4	16.3	25.9
**Smoking, %**	Daily	67.4	67.1	67.9	67.3	67.8	66.8	66.9	71.6
	Non-daily	10.9	11.5	10.0	11.3	10.0	10.9	11.0	10.3
	Stopped last year	21.7	21.4	22.1	21.5	22.2	22.3	22.0	18.1
**Type of cigarette, %**	Manufactured	47.4	51.7	41.1	50.4	39.1	50.8	44.5	37.3
	Roll-your-own	43.0	5.3	6.3	5.0	7.6	4.7	6.9	8.0
	Mix	5.7	39.0	48.9	40.3	50.6	40.2	45.8	50.5
	Unknown	3.9	4.0	3.7	4.3	2.6	4.3	2.9	4.2
**Time since most recent quit attempt, %**	< 1 month	16.1	15.7	16.6	15.1	18.7	16.1	15.6	16.8
	1 to 6 months	47.2	46.6	48.0	47.5	46.0	44.8	50.8	50.8
	> 6 months	36.8	37.7	35.4	37.3	35.3	39.1	33.7	32.4
**Unplanned quit attempt, %**		51.7	51.3	52.2	52.2	50.1	51.8	49.1	57.6
**Stopped without cutting down, %**		53.8	52.5	55.7	54.6	51.4	54.4	51.9	54.7
**Strength of urges to smoke, M (SD)**		1.75 (1.20)	1.65 (1.15)	1.89 (1.26)	1.68 (1.16)	1.94 (1.28)	1.64 (1.14)	1.82 (1.22)	2.10 (1.36)
***N*past-year quit attempts, M (SD)**		1.78 (5.17)	1.78 (5.48)	1.78 (4.68)	1.78 (5.47)	1.77 (4.21)	1.70 (5.44)	1.97 (5.51)	1.69 (1.88)

Unweighted *n* = 1956

^1^A provided third option was not used by anyone in the included sample

^2^AB: higher or intermediate managerial, administrative or professional occupation; C1: supervisory or clerical and junior managerial, administrative or professional; C2: skilled manual workers; D: semi-skilled and unskilled manual workers; E: state pensioners, casual and lowest grade workers, unemployed with state benefits only

All age groups were represented with those aged 65 and over the smallest group; just over half of the sample were men, and the full range of occupational grades was represented. Two thirds (67.4%) were daily smokers, and just over a fifth (21.7%) had stopped smoking in the last year. About half (48.7%) smoked or had smoked roll-your-own cigarettes only or as well as manufactured cigarettes. On average, respondents had made just under 2 quit attempts in the past year. Almost half (47.2%) of the most recent quit attempts had started 1 to 6 months ago, 36.8% started more than 6 months ago and the remaining 16.1% had started within the last month. About half (51.7%) of these quit attempts had been unplanned, and a similar proportion (53.8%) had stopped without cutting down. On average, respondents experienced slight to moderate urges to smoke (Table 2).

### Quit attempt triggers

Overall, the three most frequently reported triggers were ‘concern about future health’ (36.5%), ‘health problems I had at the time’ (19.7%) and ‘a decision that smoking was too expensive’ (19.0%). Further reasons with more than 5% agreement were ‘something said by friends/family (17.0%), ‘general practitioner (GP)/health professional advice’ (13.5%), ‘others’ (5.7%) and ‘knew someone else who was stopping’ (5.6%, Tables 3 and 4).

**Table 3:** Associations between mental health and triggers for quit attempts

	Concern re future health			Current health problem			Too expensive			Something said by friends/family		
	%^**1**^	_**adj**_OR, 95% CI^**2**^	***p***	%^**1**^	_**adj**_OR, 95% CI ^**2**^	***p***	%^**1**^	_**adj**_OR, 95% CI^**2**^	***p***	%^**1**^	_**adj**_OR, 95% CI^**2**^	***p***
**Ever diagnosis**												
No	36.2	Ref		17.5	Ref		18.0	Ref		16.1	Ref	
Yes	37.0	1.16, 0.95–1.41	0.15	23.5	**1.55, 1.23–1.97**	**< 0.001**	20.4	1.20, 0.94–1.52	0.14	18.4	1.21, 0.94–1.56	0.15
**Past-year treatment**												
No	36.7	Ref		17.7	Ref		18.9	Ref		16.5	Ref	
Yes	35.9	1.10, 0.88–1.38	0.40	25.4	**1.52, 1.18–1.97**	**0.001**	19.4	1.05, 0.80–1.37	0.74	18.5	1.13, 0.85–1.49	0.41
**Past-month distress**												
None	36.6	Ref		18.2	Ref		18.2	Ref		14.2	Ref	
Moderate	37.5	1.15, 0.92–1.43	0.22	20.3	1.25, 0.96–1.63	0.099	21.0	1.15, 0.88–1.49	0.30	21.5	**1.49, 1.13–1.96**	**0.005**
Serious	33.6	1.06, 0.77–1.45	0.71	25.8	**1.72, 1.21–2.43**	**0.002**	18.4	1.06, 0.73–1.54	0.77	20.2	**1.52, 1.03–2.22**	**0.034**

Unweighted *n* = 1956

Bold figures indicate that the confidence interval does not include 1

^1^Weighted % who reported each trigger, those with > 5% overall only

^2^Logistic regressions unweighted, adjusted for age, gender, occupational grade, urges to smoke, time since most recent quit attempt

**Table 4:** Associations between mental health and triggers for quit attempts continued

	GP/health professional advice			Others			Knew someone else who was stopping		
	%^**1**^	_**adj**_OR, 95% CI^**2**^	***p***	%^**1**^	_**adj**_OR, 95% CI^**2**^	***p***	%^**1**^	_**adj**_OR, 95% CI^**2**^	***p***
**Ever diagnosis**									
No	14.2	Ref		5.4	Ref		5.4	Ref	
Yes	12.4	0.86, 0.65–1.14	0.30	6.2	1.33, 0.89–2.00	0.16	5.7	1.07, 0.70–1.62	0.76
**Past-year treatment**									
No	13.5	Ref		5.7	Ref		5.4	Ref	
Yes	13.5	0.99, 0.73–1.36	0.96	5.7	1.21, 0.78–1.89	0.40	5.9	1.19, 0.75–1.89	0.46
**Past-month distress**									
None	14.8	Ref		5.5	Ref		5.7	Ref	
Moderate	10.8	0.83, 0.61–1.14	0.26	6.4	1.38, 0.89–2.13	0.155	5.4	0.86, 0.54–1.37	0.54
Serious	13.5	1.02, 0.66–1.56	0.94	5.3	1.18, 0.62–2.25	0.61	4.5	0.87, 0.44–1.73	0.70

Unweighted *n* = 1956

Bold figures indicate that the confidence interval does not include 1

^1^Weighted % who reported each trigger, those with > 5% overall only

^2^Logistic regressions unweighted, adjusted for age, gender, occupational grade, urges to smoke, time since most recent quit attempt

In adjusted analysis, respondents who have ever had a diagnosis, had past-year treatment or had serious past-month distress were more likely than those without to select ‘health problems I had at the time’. Respondents with moderate or serious distress were additionally more likely to select ‘something said by family/friends/children’ than those without distress (Tables 3 and 4). In unplanned analyses including the type of cigarette, those who have ever had a diagnosis were more likely to select ‘too expensive’ (adjusted OR = 1.29, 95% confidence interval (CI) 1.01 to 1.64, *p* = 0.041). Unadjusted logistic regression models for all variables are provided in Additional file 1, Tables S2 and S3.

The remaining reasons were selected by fewer than 4% of respondents: seeing a health warning on a cigarette packet, 3.3%; being faced with smoking restrictions, 3.1%; pregnancy, 3.1%; (just) decided to quit, 2.3%; TV advert for a nicotine replacement product, 2.2%; a significant birthday, 1.5%; government TV/radio/press advert, 1.2%; being contacted by my local National Health Service (NHS) Stop Smoking Services, 0.8%; hearing about a new stop smoking treatment, 0.7%; and attending a local stop smoking activity or event, 0.7%.

### Support used

The most popular support used was non-evidence-based (42.0%), followed by e-cigarettes (35.2%) and NRT over the counter (12.0%). Few had used prescription medication (7.9%), behavioural support (2.0%) or a combination of those two (0.9%). The last three groups were collapsed for further analyses (prescription medication and/or behavioural support, 10.8%). Respondents who have ever had a mental health diagnosis or moderate or serious past-month distress were less likely to have used NRT over the counter than those without those indicators of a mental health problem (Table 5). Respondents with past-year treatment and those with serious distress were more likely to have used prescription medication and/or behavioural support. Use of non-evidence-based support and of e-cigarettes was similar across the groups (Table 5). In unplanned analyses including the type of cigarette, those who have ever had a diagnosis were more likely to have used prescription and/or behavioural support than those without (OR = 1.37, 95% CI 1.01 to 1.86, *p* = 0.046). Unadjusted logistic regression models for all variables are provided in Additional file 1, Table S4.

**Table 5:** Associations between mental health and support used during quit attempts

	Non-evidence-based			NRT over the counter			E-cigarettes			Prescription and/or behavioural support		
	Used by, %	_**adj**_OR, 95% CI^1^	***p***	Used by, %	_**adj**_OR, 95% CI^1^	***p***	Used by, %	_**adj**_OR, 95% CI^1^	***p***	Used by, %	_**adj**_OR, 95% CI^1^	***p***
**Ever diagnosis**												
No	42.3	Ref		13.5	Ref		33.9	Ref		10.2	Ref	
Yes	41.6	0.91, 0.74–1.10	0.33	**9.8**	**0.66, 0.48–0.89**	**0.006**	37.0	1.17, 0.96–1.43	0.12	11.6	1.34, 0.99–1.82	0.061
**Past-year treatment**												
No	42.6	Ref		12.4	Ref		34.9	Ref		10.1	Ref	
Yes	40.0	0.90, 0.72–1.12,	0.34	10.9	0.83, 0.59–1.16	0.28	36.1	1.03, 0.83–1.29	0.77	**12.8**	**1.42, 1.02–1.97**	**0.039**
**Past-month distress**												
None	41.1	Ref		14.1	Ref		34.2	Ref		10.5	Ref	
Moderate	42.5	1.07, 0.86–1.32	0.57	10.2	**0.69, 0.50–0.97**	**0.030**	37.6	1.06, 0.85–1.32	0.61	9.8	1.11, 0.78–1.57	0.58
Serious	45.5	1.15, 0.85–1.56	0.36	6.1	**0.35, 0.19–0.64**	**0.001**	34.0	0.91, 0.66–1.24	0.53	**14.3**	**2.05, 1.32–3.18**	**0.001**

Weighted % who used each type of support, logistic regressions unweighted

Unweighted *n* = 1956

Bold figures indicate that the confidence interval does not include 1

^1^Adjusted for age, gender, occupational grade, abrupt quit, unplanned quit and urges to smoke. Support used is hierarchically dummy-coded

### Quit success

Overall, 18.3% of those who had started their quit attempt at least 1 month before the survey were still abstinent; this rose to 19.1% of those who had started their quit attempt at any time including less than 1 month before the survey. Unadjusted logistic regression models for all variables are provided in Additional file 1, Table S5.

For analyses including past-month distress, we could not include an interaction term for support used and distress as no respondent with serious distress had quit successfully with NRT over the counter (Additional file 1, Table S6). For ever diagnosis and past-year treatment, interaction terms were included.

In adjusted analyses, quit success was not significantly associated with ever having had a mental health diagnosis, past-year treatment or past-month distress (Table 6), with moderate evidence to support the null hypothesis compared with a modest negative association (OR = 0.65) on ever diagnosis and treatment (BFs 0.20 and 0.26) and data insensitive on past-month distress (BFs 0.70 and 0.41).

**Table 6:** Adjusted associations with quit success using three different indicators for mental health (ever diagnosis, past-year treatment, past-month distress)

	Quit > 1 month			Quit any length		
	Success rate, %^**1**^	OR, 95% CI	***p***	Success rate, %^**1**^	OR, 95% CI	***p***
**Ever diagnosis**						
No	18.3	Ref		18.8	Ref	
Yes	18.1	1.14 (0.83–1.57)	0.43	19.6	1.23 (0.94–1.62)	0.13
**Support used**						
Non-evidence-based	16.4	Ref		16.5	Ref	
NRT over the counter	14.1	1.06 (0.62–1.81)	0.84	16.7	1.32 (0.85–2.07)	0.22
E-cigarettes	21.5	**2.25 (1.59–3.18)**	**< 0.001**	23.0	**2.21 (1.64–2.98)**	**< 0.001**
Prescription and/or behavioural support	18.9	1.68 (1.00–2.84)	0.051	18.8	1.46 (0.92–2.31)	0.11
**Past-year treatment**						
No	18.3	Ref		19.2	Ref	
Yes	18.0	1.13 (0.78–1.62)	0.52	18.9	1.08 (0.80–1.48)	0.61
**Support used**^2^						
Non-evidence-based		Ref			Ref	
NRT over the counter		1.05 (0.61–1.80)	0.86		1.31 (0.84–2.05)	0.24
E-cigarettes		**2.24 (1.58–3.17)**	**< 0.001**		**2.21 (1.64–2.98)**	**< 0.001**
Prescription and/or behavioural support		1.68 (1.00–2.84)	0.051		1.48 (0.93–2.34)	0.099
**Past-month distress**						
No	19.9	Ref		20.9	Ref	
Moderate	16.2	0.88 (0.61–1.27)	0.49	17.1	0.89 (0.65–1.21)	0.46
Serious	15.3	1.11 (0.66–1.87)	0.69	14.8	0.89 (0.57–1.40)	0.61
**Support used**^2^						
Non-evidence-based		Ref			Ref	
NRT over the counter		1.06 (0.62–1.82)	0.84		1.29 (0.83–2.03)	0.26
E-cigarettes		**2.25 (1.59–3.18)**	**< 0.001**		**2.21 (1.64–2.97)**	**< 0.001**
Prescription and/or behavioural support		**1.69 (1.01–2.86)**	**0.047**		1.50 (0.95–2.38)	0.085

Unweighted *n* = 1956 for quit any length, *n* = 1639 for quit success > 1 month

Adjusted for age, gender, occupational grade, urges to smoke, time since quit attempt started, number of quit attempts in the past year and type of cigarette

*NRT* nicotine replacement therapy, *OTC* over-the-counter (without prescription)

^1^Weighted % with the relevant outcome, logistic regressions unweighted

^2^Success rates as shown in first section of table

Compared with non-evidence-based support, e-cigarettes were positively associated with quit success (both of at least 1 month and of any length). The association was similar across the three models that separately included the different indicators of mental health (Table 6). Prescription medication and/or behavioural support was also significantly associated with greater quit success of at least 1 month when including past-month distress (OR 1.69). The association was similar but non-significant in models including ever diagnosis or past-year treatment instead of distress (OR 1.68 and OR 1.68). Across all models, BFs revealed that a positive association between prescription medication and/or behavioural support and quit success was more likely than the null. Depending on the outcome (abstinence of at least 1 month or any length) and the magnitude of the expected positive association, BFs ranged from 1.35 (anecdotal evidence) to 3.82 (moderate evidence). NRT over the counter was not significantly associated with quit success, and the BF (0.96) indicated anecdotal evidence for the null hypothesis. There were no significant interactions between ever diagnosis and support used (*p* = 0.96) or between past-year treatment and support used (*p* = 0.99) in relation to quit success. Associations between quit success and mental health indicators or support used remained similar with and without interactions included.

## Discussion

Among smokers with and without mental health problems, the most frequent triggers of quit attempts were concerns about current and future health, cost of smoking, something said by friends/family and advice from a GP or health professional. There were small differences in that current health and comments from friends/family were a more frequent trigger for those with mental health problems. There was some indication that during a quit attempt, those with mental health problems were less likely to have used NRT over the counter and more likely to have used prescription medication or behavioural support. There was no difference in the use of e-cigarettes during a quit attempt and e-cigarettes conferred a benefit over non-evidence-based support. Smokers with mental health problems were as likely to be successful in their quit attempt as smokers without mental health problems, and type of support was not differentially associated with success in those with and without mental health problems.

Quit attempt triggers appear to have changed over time. Compared with a study which used data from 2009 to 2012 from the same survey [35], concerns about future health (2009–2012, 20.6%; 2016–2017, 36.1%) and current health problems (2009–2012, 11.8%; 2016–2017, 20.8%) appear to have become more common. In the earlier study, advice from a GP or health professional was the top reason (24.5%); this had fallen to fifth place in the more recent data (14.8%) overall and was no more common among those with mental health problems.

The rating of triggers highlights where changes could help increase quit attempts. The decline in the relevance of healthcare professionals’ advice may negatively affect quitting among smokers with mental health problems. Brief advice has been shown to improve long-term quit rates in the general population of smokers [37, 38], and guidelines for healthcare professionals on providing very brief advice on smoking at every opportunity [39] may need to be emphasised. The present results also indicate that media campaigns and smoking cessation services appear not to be widely cited by smokers, which may partly be due to a lack of funding in the years of data collection [40, 41]. To help reduce inequalities, campaigns should be developed and targeted to be particularly effective for smokers with mental health problems. Stop smoking services used to be available across England and the services were able to help reduce inequalities [42]; however, services have been reduced considerably in recent years [43]. Evidence-based support in the form of medication and behavioural support provided by trained practitioners needs to be available for all those who need them [44]. Having professionals who can lead on supporting smokers with mental health problems may also be beneficial [45].

It is encouraging that smokers with recent treatment for a mental health problem or with distress were more likely to have used support in the form of prescription medication or behavioural support which has been shown to be effective in the general population and those with severe mental health problems [21, 22]. This will be partly because smokers who have recently been treated for a mental health problem are likely to have had more frequent contact with healthcare providers than other smokers which increases the overall chance of being offered treatment [46].

Reviews of trials found varenicline and bupropion to be efficacious for smoking cessation in smokers with mental health problems [21, 22], and an analysis of Smoking Toolkit Study data collected from 2006 to 2018 also found varenicline to be associated with higher success rates [30]. In the present study, there was anecdotal to moderate evidence of an effect of prescription medication (which included varenicline, bupropion and NRT). Grouping different prescription medication options and grouping them with different levels of behavioural support may have attenuated effects. As in the present study, the previous survey analysis found e-cigarettes to be associated with increased success [30].

Systematic reviews indicate that, with the exception of stop smoking services that can compensate for lower quit rates in disadvantaged populations through increased reach, individual-level behavioural and pharmacological smoking cessation interventions often do not reduce socio-economic inequalities in smoking [42, 47]. Evidence on interventions that help reduce inequalities related to mental health is sparse as is evidence on the effectiveness of e-cigarettes for smoking cessation or reduction among smokers with mental health problems. A recent systematic review identified no controlled trials and only four small single-group pre-post studies which suggested some benefit [48]. The present findings provide some evidence on the likely impact of e-cigarettes on inequalities caused by smoking. That they were the type of support associated with the greatest increase in success rates, used to a similar extent and with similar success by smokers with and without mental health problems, indicates that e-cigarettes used in quit attempts currently are more likely to positively affect inequalities than other smoking cessation interventions. However, they may have further potential to reduce inequalities if their reach or effectiveness among those with mental health problems can be increased. Better reach could be supported by improved understanding in the general public and among health professionals that e-cigarettes provide a less harmful alternative to smoking [49, 50] and are effective for quitting smoking [51, 52], particularly in combination with behavioural support as provided by stop smoking services [51, 53].

The present study addresses a lack of evidence on ways to improve smoking cessation among the underserved but large group of smokers with mental health problems. It is the first study to provide population-level evidence on triggers of quit attempts; type of support used, including e-cigarettes; and outcomes of quit attempts in smokers with mental health problems compared with those without.

Limitations of the study include that the data collection was cross-sectional and information therefore retrospective. As a household survey, people too unwell to participate or in institutions were excluded, so the findings may not generalise to those experiencing severe mental health problems. Due to the sampling strategy used, a response rate cannot be calculated; however, samples obtained have been shown to be similar in sociodemographic composition, smoking rates and consumption to the general population [24, 54]. Smoking abstinence was not biochemically validated, but previous work has shown that accurate estimates of smoking prevalence can be derived from self-report in population surveys [55–57]. The proportion of respondents who had used prescription medication or behavioural support was too small to assess the effectiveness of different medications or the separate effect of behavioural support. Although mental health status was self-reported, the use of three different indicators of mental health strengthens confidence in the findings. The study used a long-standing data source with established analysis approaches and a pre-registered analysis plan.

## Conclusions

In conclusion, smokers with mental health problems frequently reported that quit attempts were triggered by concerns around health, costs of smoking and comments from those close to them. Smokers with mental health problems were more likely to have used evidence-based support in the form of prescription medication or behavioural support and not NRT without prescription. E-cigarettes were associated with increased success and they were used similarly across those with and without mental health problems, indicating that improved uptake of e-cigarettes for smoking cessation among smokers with mental health problems could help address inequalities.

## Supplementary information

**Additional file 1: Table S1.** Weighted prevalence of individual diagnoses. **Table S2.** Unadjusted associations with triggers. **Table S3.** Unadjusted associations with triggers continued. **Table S4.** Unadjusted associations with support used. **Table S5.** Unadjusted associations with quit success. **Table S6.** Weighted success rates by support used and mental health status.

## Data Availability

All authors have had access to the data. The datasets analysed during the current study are available from the corresponding author on reasonable request.

## Abbreviations

BF: Bayes factor
CI: Confidence interval
GP: General practitioner
NHS: National Health Service
NICE: National Institute for Health and Care Excellence
NRT: Nicotine replacement therapy
OR: Odds ratio
UK: United Kingdom
US: United States of America
