# Supplementary material for: Mental health and smoking cessation—a population survey in England
Source: BMC Med. 2020 Jun 25;18:161. doi: 10.1186/s12916-020-01617-7 (PMC7315517; doi:10.1186/s12916-020-01617-7)
Supplement: Supplementary file 1 — Additional file 1: Table S1. Weighted prevalence of individual diagnoses. Table S2. Unadjusted associations with triggers. Table S3. Unadjusted associations with triggers continued. Table S4. Unadjusted associations with support used. Table S5. Unadjusted associations with quit success. Table S6. Weighted success rates by support used and mental health status. [file 12916_2020_1617_MOESM1_ESM.docx]

## Mental health and smoking cessation – a population survey in England

### Leonie S. Brose, Jamie Brown, Ann McNeill

## Supplementary material

**Table S1. Weighted prevalence of individual diagnoses**

| **Diagnosis** | **Prevalence, %** |
| --- | --- |
| Depression | 30.1 |
| Anxiety | 23.5 |
| Panic Disorder or Phobia | 6.4 |
| Post-Traumatic Stress Disorder (PTSD) | 3.6 |
| Eating Disorder | 3.1 |
| Alcohol Misuse or Dependence | 2.9 |
| Obsessive Compulsive Disorder | 2.5 |
| Personality Disorder | 2.4 |
| Drug Use or Dependence | 2.3 |
| Attention Deficit Hyperactivity Disorder (ADHD) | 2.0 |
| Psychosis | 1.7 |
| Problem Gambling | 0.5 |

**Table S2. Unadjusted associations with triggers**

|  | **OUTCOME** | | | | | | | |
| --- | --- | --- | --- | --- | --- | --- | --- | --- |
|  | **Concern re future health** | | **Current health problem** | | **Too expensive** | | **Something said by friends/family** | |
|  | **OR, 95% CI** | **p** | **OR, 95% CI** | **p** | **OR, 95% CI** | **p** | **OR, 95% CI** | **p** |
| **Ever diagnosis** | 1.02 (0.84-1.23) | 0.87 | 1.52 (1.22-1.89) | <0.001 | 1.13 (0.90-1.42) | 0.28 | 1.20 (0.94-1.53) | 0.13 |
| **Past-year treatment** | 0.93 (0.75-1.15) | 0.50 | 1.55 (1.22-1.96) | <0.001 | 0.98 (0.76-1.26) | 0.87 | 1.11 (0.85-1.45) | 0.45 |
| **Past-month distress** |  | 0.51 |  | 0.023 |  | 0.52 |  | 0.003 |
| Moderate | 1.08 (0.87-1.33) | 0.49 | 1.13 (0.88-1.45) | 0.34 | 1.16 (0.90-1.49) | 0.26 | 1.53 (1.18-2.01) | 0.002 |
| Serious | 0.89 (0.66-1.20) | 0.45 | 1.57 (1.14-2.18) | 0.006 | 1.02 (0.71-1.46) | 0.91 | 1.51 (1.05-2.17) | 0.028 |
| **Age** (16-24 ref) |  | 0.31 |  | <0.001 |  | 0.80 |  |  |
| 25-34 | 1.04 (0.79-1.38) | 0.77 | 1.01 (0.68-1.49) | 0.97 | 0.80 (0.57-1.12) | 0.20 |  | <0.001 |
| 35-44 | 1.14 (0.85-1.53) | 0.39 | 1.61 (1.10-2.36) | 0.015 | 0.84 (0.59-1.20) | 0.33 | 1.44 (1.01-2.04) | 0.044 |
| 45-54 | 1.13 (0.84-1.54) | 0.42 | 1.89 (1.28-2.77) | 0.001 | 0.79 (0.55-1.15) | 0.22 | 1.26 (0.86-1.83) | 0.24 |
| 55-64 | 0.82 (0.58-1.15) | 0.25 | 3.22 (2.18-4.76) | <0.001 | 0.82 (0.55-1.23) | 0.4 | 0.76 (0.50-1.16) | 0.20 |
| 65+ | 0.84 (0.57-1.23) | 0.36 | 2.75 (1.79-4.22) | <0.001 | 0.81 (0.52-1.27) | 0.36 | 0.56 (0.32-1.00) | 0.048 |
| **Women** (men ref) | 0.69 (0.57-0.83) | <0.001 | 1.34 (1.07-1.66) | 0.010 | 0.94 (0.75-1.17) | 0.58 | 0.84 (0.66-1.07) | 0.15 |
| **Occupational grade** (AB ref) |  | <0.001 |  | 0.025 |  | 0.027 |  | 0.33 |
| C1 | 0.72 (0.54-0.94) | 0.017 | 1.23 (0.86-1.75) | 0.26 | 1.23 (0.88-1.72) | 0.22 | 0.74 (0.52-1.05) | 0.096 |
| C2 | 0.54 (0.41-0.73) | <0.001 | 1.38 (0.95-2.00) | 0.087 | 0.87 (0.60-1.25) | 0.45 | 0.82 (0.56-1.18) | 0.28 |
| D | 0.57 (0.41-0.78) | <0.001 | 1.21 (0.81-1.81) | 0.35 | 0.93 (0.62-1.37) | 0.70 | 0.70 (0.47-1.06) | 0.097 |
| E | 0.37 (0.26-0.52) | <0.001 | 1.86 (1.26-2.75) | 0.002 | 0.69 (0.45-1.06) | 0.093 | 0.67 (0.44-1.04) | 0.072 |
| **Strength of urges** | 0.97 (0.90-1.05) | 0.45 | 1.19 (1.09-1.30) | <0.001 | 1.03 (0.94-1.13) | 0.57 | 1.03 (0.93-1.14) | 0.564 |
| **Quit attempt started >6 months ago** (<6 months ref) | 0.90 (0.74-1.09) | 0.26 | 1.09 (0.87-1.37) | 0.43 | 0.69 (0.54-0.88) | 0.002 | 0.94 (0.73-1.21) | 0.654 |

Unweighted n=1,956

**Table S3. Unadjusted associations with triggers continued**

|  | **OUTCOME** | | | | | |
| --- | --- | --- | --- | --- | --- | --- |
|  | **GP/health professional advice** | | **Other** | | **Knew someone else who was stopping** | |
|  | **OR, 95% CI** | **p** | **OR, 95% CI** | **p** | **OR, 95% CI** | **p** |
| **Ever diagnosis** | 0.83 (0.64-1.08) | 0.16 | 1.32 (0.90-1.95) | 0.16 | 0.95 (0.64-1.42) | 0.82 |
| **Past-year treatment** | 0.98 (0.73-1.31) | 0.87 | 1.19 (0.78-1.82) | 0.43 | 1.00 (0.64-1.56) | 0.99 |
| **Past-month distress** |  | 0.131 |  | 0.39 |  | 0.82 |
| Moderate | 0.73 (0.54-0.99) | 0.045 | 1.34 (0.88-2.06) | 0.17 | 0.91 (0.58-1.42) | 0.67 |
| Serious | 0.89 (0.59-1.33) | 0.55 | 1.14 (0.61-2.11) | 0.68 | 0.83 (0.43-1.60) | 0.58 |
| **Age** (16-24 ref) |  | <0.001 |  | 0.23 |  | 0.070 |
| 25-34 | 1.39 (0.85-2.25) | 0.188 | 1.04 (0.59-1.83) | 0.90 | 0.84 (0.48-1.46) | 0.54 |
| 35-44 | 1.71 (1.04-2.79) | 0.033 | 0.78 (0.41-1.48) | 0.45 | 0.99 (0.56-1.74) | 0.97 |
| 45-54 | 2.25 (1.39-3.65) | 0.001 | 1.32 (0.74-2.38) | 0.35 | 0.69 (0.37-1.31) | 0.26 |
| 55-64 | 3.72 (2.30-6.02) | <0.001 | 0.68 (0.32-1.46) | 0.33 | 0.17 (0.05-0.56) | 0.004 |
| 65+ | 5.21 (3.16-8.57) | <0.001 | 0.47 (0.17-1.24) | 0.13 | 0.64 (0.28-1.44) | 0.28 |
| **Women** (men ref) | 1.16 (0.90-1.50)) | 0.24 | 1.07 (0.72-1.57) | 0.75 | 0.77 (0.52-1.15) | 0.20 |
| **Occupational grade** (AB ref) |  | 0.21 |  | 0.55 |  | 0.015 |
| C1 | 0.96 (0.64-1.44) | 0.84 | 1.33 (0.74-2.38) | 0.34 | 1.15 (0.63-2.09) | 0.64 |
| C2 | 1.20 (0.79-1.82) | 0.39 | 0.93 (0.49-1.78) | 0.83 | 1.53 (0.84-2.78) | 0.16 |
| D | 1.02 (0.65-1.62) | 0.93 | 0.96 (0.47-1.93) | 0.90 | 0.83 (0.40-1.72) | 0.62 |
| E | 1.48 (0.95-2.30) | 0.082 | 0.85 (0.41-1.79) | 0.68 | 0.32 (0.12-0.88) | 0.027 |
| **Strength of urges** | 1.12 (1.01-1.24) | 0.033 | 0.89 (0.75-1.04) | 0.15 | 1.02 (0.87-1.20) | 0.81 |
| **Quit attempt started >6 months ago** (<6 months ref) | 1.02 (0.79-1.33) | 0.87 | 0.93 (0.62-1.40) | 0.73 | 0.96 (0.64-1.44) | 0.84 |

Unweighted n=1,956

**Table S4. Unadjusted associations with support used**

|  | **OUTCOME** | | | | | | | |
| --- | --- | --- | --- | --- | --- | --- | --- | --- |
|  | **Non-evidence-based** | | **NRT over the counter** | | **E-cigarettes** | | **Prescription and / or Behavioural support** | |
|  | **OR, 95% CI** | **p** | **OR, 95% CI** | **p** | **OR, 95% CI** | **p** | **OR, 95% CI** | **p** |
| **Ever diagnosis** | 0.90 (0.75-1.08) | 0.26 | 0.67 (0.51-0.90) | 0.007 | 1.22 (1.01-1.48) | 0.036 | 1.23 (0.92-1.63) | 0.156 |
| **Past-year treatment** | 0.86 (0.70-1.06) | 0.154 | 0.86 (0.63-1.18) | 0.34 | 1.10 (0.89-1.36) | 0.38 | 1.35 (0.99-1.83) | 0.055 |
| **Past-month distress** |  | 0.60 |  | <0.001 |  | 0.40 |  | 0.064 |
| Moderate | 1.07 (0.87-1.31) | 0.54 | 0.68 (0.50-0.94) | 0.019 | 1.16 (0.94-1.43) | 0.18 | 0.92 (0.66-1.29) | 0.63 |
| Serious | 1.14 (0.86-1.52) | 0.36 | 0.35 (0.20-0.63) | <0.001 | 1.03 (0.77-1.39) | 0.84 | 1.53 (1.03-2.29) | 0.036 |
| **Age** (16-24 ref) |  | <0.001 |  | <0.001 |  | 0.009 |  | <0.001 |
| 25-34 | 0.64 (0.49-0.84) | 0.001 | 2.29 (1.36-3.88) | 0.002 | 1.02 (0.77-1.36) | 0.86 | 2.29 (1.21-4.30) | <0.001 |
| 35-44 | 0.53 (0.39-0.70) | <0.001 | 2.98 (1.76-5.06) | <0.001 | 0.92 (0.68-1.24) | 0.59 | 3.68 (1.98-6.84) | 0.010 |
| 45-54 | 0.50 (0.37-0.68) | <0.001 | 2.66 (1.54-4.59) | <0.001 | 0.90 (0.66-1.22) | 0.50 | 4.74 (2.56-8.75) | <0.001 |
| 55-64 | 0.45 (0.33-0.63) | <0.001 | 3.66 (2.11-6.35) | <0.001 | 0.77 (0.55-1.08) | 0.13 | 5.44 (2.89-10.21) | <0.001 |
| 65+ | 0.66 (0.46-0.94) | 0.021 | 3.18 (1.74-5.79) | <0.001 | 0.49 (0.32-0.74) | 0.001 | 6.18 (3.21-11.89) | <0.001 |
| **Women** (men ref) | 0.91 (0.76-1.08) | 0.28 | 1.02 (0.78-1.33) | 0.91 | 0.98 (0.81-1.18) | 0.79 | 1.33 (1.00-1.77) | 0.049 |
| **Occupational grade** (AB ref) |  | 0.008 |  | 0.96 |  | 0.011 |  | 0.46 |
| C1 | 1.15 (0.88-1.51) | 0.31 | 1.01 (0.67-1.53) | 0.96 | 1.00 (0.74-1.34) | 0.98 | 0.70 (0.46-1.07) | 0.099 |
| C2 | 0.74 (0.55-0.99) | 0.040 | 0.96 (0.62-1.49) | 0.85 | 1.53 (1.13-2.06) | 0.006 | 0.84 (0.54-1.31) | 0.44 |
| D | 0.82 (0.60-1.12) | 0.21 | 0.99 (0.62-1.60) | 0.97 | 1.27 (0.91-1.77) | 0.154 | 0.97 (0.61-1.54) | 0.88 |
| E | 0.93 (0.68-1.28) | 0.66 | 1.15 (0.71-1.84) | 0.58 | 1.14 (0.81-1.60) | 0.46 | 0.78 (0.47-1.29) | 0.33 |
| **Abrupt quit** | 1.39 (1.16-1.67) | <0.001 | 0.79 (0.60-1.03) | 0.086 | 0.94 (0.78-1.13) | 0.50 | 0.67 (0.50-0.89) | 0.005 |
| **Unplanned quit** | 1.76 (1.47-2.12) | <0.001 | 0.61 (0.46-0.80) | <0.001 | 0.91 (0.76-1.10) | 0.35 | 0.51 (0.38-0.69) | <0.001 |
| **Strength of urges** | 0.79 (0.73-0.85) | <0.001 | 1.14 (1.02-1.28) | 0.019 | 1.10 (1.02-1.19) | 0.013 | 1.22 (1.08-1.37) | 0.001 |
| **Type of cigarette** (Manufactured ref) | 1.12 (0.93-1.34) | 0.22 | 1.00 (0.76-1.31) | 0.98 | 1.04 (0.86-1.26) | 0.66 | 0.69 (0.52-0.92) | 0.011 |

Unweighted n=1,956

**Table S5. Unadjusted associations with quit success**

|  | **OUTCOME** | | | |
| --- | --- | --- | --- | --- |
|  | **Quit >1month** | | **Quit any length** | |
|  | **OR, 95% CI** | **p** | **OR, 95% CI** | **p** |
| **Support** (non-evidence-based ref) |  | **0.027** |  | **0.004** |
| NRT over the counter | 0.77 (0.48-1.21) | 0.25 | 0.91 (0.61-1.35) | 0.63 |
| E-cigarettes | **1.38 (1.04-1.83)** | **0.027** | **1.50 (1.16-1.93)** | **0.002** |
| Prescription/Support | 0.95 (0.62-1.47) | 0.82 | 0.95 (0.64-1.43) | 0.81 |
| **Ever diagnosis** | 0.96 (0.74-1.24) | 0.75 | 1.02 (0.81-1.29) | 0.85 |
| **Past-year treatment** | 0.93 (0.70-1.25) | 0.64 | 0.92 (0.71-1.19) | 0.53 |
| **Past-month distress** |  | **0.045** |  | **0.029** |
| Moderate | **0.69 (0.52-0.94)** | **0.017** | **0.73 (0.56-0.96)** | **0.022** |
| Serious | 0.78 (0.51-1.18) | 0.234 | 0.71 (0.48-1.04) | 0.076 |
| **Age** (16-24 ref) |  | 0.149 |  | 0.427 |
| 25-34 | 1.28 (0.86-1.90) | 0.23 | 1.00 (0.71-1.42) | 0.997 |
| 35-44 | 1.03 (0.67-1.57) | 0.91 | 0.85 (0.59-1.24) | 0.41 |
| 45-54 | 1.01 (0.65-1.57) | 0.98 | 0.90 (0.61-1.32) | 0.58 |
| 55-64 | 1.25 (0.78-2.00) | 0.36 | 1.15 (0.77-1.72) | 0.49 |
| 65+ | 1.78 (1.10-2.86) | 0.018 | 1.31 (0.85-2.03) | 0.21 |
| **Women** (men ref) | 0.94 (0.73-1.21) | 0.62 | 0.91 (0.72-1.14) | 0.39 |
| **Occupational grade** (AB ref) |  | 0.53 |  | 0.43 |
| C1 | 1.07 (0.73-1.57) | 0.73 | 1.07 (0.75-1.51) | 0.72 |
| C2 | 1.09 (0.73-1.63) | 0.68 | 1.01 (0.69-1.46) | 0.98 |
| D | 1.23 (0.79-1.90) | 0.36 | 1.24 (0.84-1.84) | 0.27 |
| E | 0.81 (0.50-1.31) | 0.39 | 0.83 (0.54-1.27) | 0.40 |
| **Strength of urges** | **0.28 (0.24-0.32)** | **<0.001** | **0.36 (0.32-0.41)** | **<0.001** |
| **Quit attempt started >6 months ago** (<6 months ref) | **1.71 (1.32-2.20)** | **<0.001** | **1.44 (1.15-1.82)** | **0.002** |
| **Number quit attempts** | **0.74 (0.61-0.88)** | **0.001** | **0.70 (0.60-0.82)** | **<0.001** |
| **Type of cigarette** (Manufactured ref) | 1.01 (0.78-1.30) | 0.95 | 1.05 (0.83-1.31) | 0.70 |

Unweighted n=1,956 for quit any length, n=1,639 for quit success >1 month

**Table S6. Weighted success rates by support used and mental health status**

|  | **Ever diagnosis** | | **Past-year treatment** | | **Past-month distress** | | |
| --- | --- | --- | --- | --- | --- | --- | --- |
|  | **no** | **yes** | **no** | **yes** | **none** | **moderate** | **serious** |
| **Quit success >1month** |  |  |  |  |  |  |  |
| **By type of support** |  |  |  |  |  |  |  |
| Nothing, self-help and others | 16.0 | 17.0 | 16.7 | 15.6 | 17.3 | 14.5 | 16.8 |
| NRT over the counter | 13.0 | 16.4 | 13.8 | 15.2 | 16.0 | 12.8 | 0 |
| E-cigarettes | 23.1 | 19.0 | 21.8 | 20.6 | 24.3 | 16.8 | 19.7 |
| Prescription and/or Behavioural support | 17.6 | 21.0 | 18.6 | 20.0 | 19.2 | 24.5 | 7.1 |
| **Quit success any length** |  |  |  |  |  |  |  |
| **By type of support** |  |  |  |  |  |  |  |
| Nothing, self-help and others | 15.7 | 17.5 | 16.4 | 17.0 | 16.9 | 16.0 | 15.3 |
| NRT over the counter | 14.8 | 20.7 | 15.6 | 20.3 | 19.2 | 13.3 | 0 |
| E-cigarettes | 24.3 | 21.5 | 23.7 | 21.0 | 26.3 | 17.6 | 20.5 |
| Prescription and/or Behavioural support | 18.0 | 19.6 | 19.9 | 17.4 | 20.5 | 24.1 | 5.6 |

Unweighted n=1,956 for quit any length, n=1,639 for quit success >1 month
